# The effect of photobiomodulation on the brain during wakefulness and sleep

**DOI:** 10.3389/fnins.2022.942536

**Published:** 2022-07-28

**Authors:** Cecile Moro, Audrey Valverde, Marjorie Dole, Jaimie Hoh Kam, Catherine Hamilton, Ann Liebert, Brian Bicknell, Alim-Louis Benabid, Pierre Magistretti, John Mitrofanis

**Affiliations:** ^1^FDD and CEA-LETI, Clinatec, Université Grenoble Alpes, Grenoble, France; ^2^Well Red Pty Ltd., Launceston, TAS, Australia; ^3^Governance and Research Department, Sydney Adventist Hospital, Sydney, NSW, Australia; ^4^Faculty of Health Sciences, Australian Catholic University, Sydney, NSW, Australia; ^5^Biological and Environmental Science and Engineering Division, King Abdullah University of Science and Technology, Thuwal, Saudi Arabia; ^6^Institute of Ophthalmology, University College London, London, United Kingdom

**Keywords:** glymphatics, arousal, red, non-pharmacological, infrared

## Abstract

Over the last seventy years or so, many previous studies have shown that photobiomodulation, the use of red to near infrared light on body tissues, can improve central and peripheral neuronal function and survival in both health and in disease. These improvements are thought to arise principally from an impact of photobiomodulation on mitochondrial and non-mitochondrial mechanisms in a range of different cell types, including neurones. This impact has downstream effects on many stimulatory and protective genes. An often-neglected feature of nearly all of these improvements is that they have been induced during the state of wakefulness. Recent studies have shown that when applied during the state of sleep, photobiomodulation can also be of benefit, but in a different way, by improving the flow of cerebrospinal fluid and the clearance of toxic waste-products from the brain. In this review, we consider the potential differential effects of photobiomodulation dependent on the state of arousal. We speculate that the effects of photobiomodulation is on different cells and systems depending on whether it is applied during wakefulness or sleep, that it may follow a circadian rhythm. We speculate further that the arousal-dependent photobiomodulation effects are mediated principally through a biophoton – ultra-weak light emission – network of communication and repair across the brain.

## Introduction

The brain has two very distinct global states of activity, wakefulness and sleep; it becomes quite a different functional organ in either state. In the state of wakefulness, the brain is in a conscious mode, being receptive to, and interactive with, the environment. It is occupied with orchestrating executive function, that is, attention, perception, cognition, memory and skilled movements. In the state of sleep, the brain is in an unconscious, but arousable mode and is far less receptive to the environment. In this state, the brain assumes a house-keeping function, disposing of all the metabolic debris and waste-products that have accumulated during the day before they reach toxic levels. This fluid-mediated detoxification is under circadian control (Iliff et al., 2012; Aspelund et al., 2015; Eugene and Masiak, 2015; Jessen et al., 2015; Louveau et al., 2015, 2017; Brodziak et al., 2018; Plog and Nedergaard, 2018; Hablitz et al., 2020; Mestre et al., 2020; Nedergaard and Goldman, 2020; Reddy and van der Werf, 2020; Yan et al., 2021).

Many previous studies have shown that photobiomodulation, the application of specific wavelengths of light (∼λ = 600–1000) to body tissues, has a major effect on the brain, influencing neuronal function and survival in both health and disease. That photons can stimulate a chemical change in neurones, that light energy can be converted to metabolic energy with a subsequent influence on the function and survival of the neurones. Photobiomodulation is not a targeted treatment; it can potentially help any neurone in distress, whether affected by neurodegenerative disease, psychiatric illness, or traumatic injury (Muili et al., 2012; Cassano et al., 2015; Naeser and Hamblin, 2015; Hamblin, 2016; Mitrofanis, 2019; Figueiro Longo et al., 2020; Naeser et al., 2020). Although not stated explicitly in the bulk of these previous studies, many – if not all – of the photobiomodulation treatments were undertaken during daylight hours, when the experimental animals or human subjects were in a state of wakefulness. There are, however, recent indications that photobiomodulation, when applied during the state of sleep, has a somewhat different effect; that during this state, photobiomodulation stimulates the house-keeping function of the brain, by improving the clearance of fluid-filled waste-products and debris from the brain and into the lymphatic system (Semyachkina-Glushkovskaya et al., 2021b).

In the review that follows, we will explore what is known currently of the impact of photobiomodulation on the brain in the state of wakefulness and in the state of sleep. We will then offer the speculation that photobiomodulation may have a different effect on brain function depending on what state it is in, whether wakefulness or sleep, that it may follow a circadian rhythm. Finally, we will consider the key issue of why photobiomodulation has an impact on brain function in the first place and speculate further that biophotons, the ultra-weak endogenous light emitted by cells, may form the key link between the two distinct, arousal-dependent, effects of photobiomodulation.

## Photobiomodulation and wakefulness

In almost all previous reports on the impact of photobiomodulation on body tissues, stretching back nearly seventy years, the treatment has been applied when the experimental animals or human subjects were in the state of wakefulness. These reports have shown many beneficial effects of photobiomodulation, on the functional activity of neurones in health and disease, including the protection of neurones in disease. There have also been many previous studies exploring the photobiomodulation-induced mechanisms that underpin these effects on neuronal function and survival. These issues will be considered, in turn, below.

### Photobiomodulation influences functional activity

Most – if not all – of the reports that have examined the functional activity of neurones in the brain after application of photobiomodulation have been from an external, transcranial device. The light issued from such devices has been shown by many previous studies to penetrate, at the very least as far as the cerebral cortex, approximately ten to fifteen millimeters beneath the cranial surface (see Hamblin, 2016; Mitrofanis, 2019). Indeed, transcranial photobiomodulation to normal, healthy humans – both young and older – has been reported to improve high-level cognitive functions, in terms of reaction times or performances to a range of learning and memory retrieval tasks (Barrett and Gonzalez-Lima, 2013; Gonzalez-Lima and Barrett, 2014; Blanco et al., 2017a,b; Grover et al., 2017; Jahan et al., 2019).

With increasing momentum recent reports, using a range of biological measures, have shown that transcranial photobiomodulation can influence brain and, in particular, cortical activity. After application of photobiomodulation to the motor cortex in healthy individuals, transcranial magnetic stimulation-induced hand motor-evoked potentials are very much reduced in size (Konstantinović et al., 2013). Electroencephalography (EEG) studies have reported that photobiomodulation influences the resting power spectrum of the different brain waves considerably, with increases evident in the α, β, and γ waves, but decreases in the δ and θ ones (Vargas et al., 2017; Wang et al., 2017, 2019, 2021; Jahan et al., 2019; Zomorrodi et al., 2019; Shan et al., 2021). With fMRI (functional magnetic resonance imaging), photobiomodulation has been found to affect brain activity, in particular after undertaking a certain task (i.e., task-positive), such as finger-tapping (El Khoury et al., 2019) or verbal memory (Vargas et al., 2017). For both the finger-tapping and verbal memory tasks, the overall effect of photobiomodulation was to suppress or reduce activity in the particular cortical areas activated by these tasks (Vargas et al., 2017; El Khoury et al., 2019). The effect of photobiomodulation on brain activity has been shown to be due to a metabolic influence through the activation of cytochrome oxidase c and an increase of hemoglobin oxygenation (Saucedo et al., 2021), rather than a thermal one (Dmochowski et al., 2020; Wang et al., 2021).

In addition, photobiomodulation has been reported to influence the global, large scale networks of the brain (Ghaderi et al., 2021). These large scale networks involve the coordination and intercommunication of a number of different regions of the cortical areas involved in the processing and integration of information that is necessary to generate a number of higher-order cognitive functions, such as perception, attention, memory and emotion. One of these networks, the so-called default mode network, is relatively recently described as cortical areas that show synchronous activity when individuals are seemingly at rest, not engaged in any specific mental task (Raichle, 2015). This network appears most active when individuals have internal thoughts, such as daydreaming, recalling memories, envisioning the future and mind-wandering (Raichle, 2015). While these cortical areas show elevated activity when an individual is at rest, their activity lowers when the individual is engaged in a particular task, such as focusing attention on something in the external or internal (e.g., meditation) environments (Raichle, 2015), or in a given cognitive task. Such “focusing” by an individual, appears to deactivate the default mode network, or parts thereof, so that the various task-related networks can operate (Raichle, 2015). Indeed, it is thought that individuals who cannot deactivate this network when performing a task will perform the task more poorly (Anticevic et al., 2012). A failure to deactivate the default mode network has also been reported in a number of in neurological disorders, including Alzheimer’s disease; in these cases, it has been suggested that there is an impaired ability to switch neural activities from “default” to “active and engaged” mode (Hafkemeijer et al., 2014).

In relation to the effect of photobiomodulation, it appears to influence the functional connectivity of large scale networks, particularly the default mode network (Naeser et al., 2019). In healthy subjects, transcranial photobiomodulation reduces activation during a finger-tapping task as well as resting connectivity strengths locally in parts of the default mode network (El Khoury et al., 2019). In patients suffering from either chronic stroke or Alzheimer’s disease, both of which have abnormally functioning networks, photobiomodulation could strengthen and influence functional connectivity within the default mode network itself, together with its connectivity with other networks, for example the salience and central executive networks (Chao, 2019; Naeser et al., 2019). In essence, in these damaged and/or diseased states, photobiomodulation may help correct the imbalance of functional connectivity, restoring the connectivity between cortical areas to “normal” levels (Saltmarche et al., 2017; Chao, 2019; Naeser et al., 2019; Zomorrodi et al., 2019; Spera et al., 2021). These improvements in functional connectivity manifest in improvements in cognition and memory in, for example, Alzheimer’s disease patients (Lim, 2014; Berman et al., 2017; Saltmarche et al., 2017; Chao, 2019; Baik et al., 2021).

Finally, there are several previous studies showing that transcranial photobiomodulation increases functional activity in both young and in particular, older healthy adults, by elevating cytochrome oxidase c oxidation, together with hemoglobin oxygenation (Wang et al., 2017; Saucedo et al., 2021).

### Photobiomodulation induces neuroprotection

Photobiomodulation has been shown, not only to influence the functional activity of neurones across the brain, but also to improve their survival against damage or disease. Such improvements, referred to commonly as disease-modifying or neuroprotective outcomes, have been reported in a range of animal models of disease or trauma, including; retinal disease (Eells et al., 2004; Natoli et al., 2010; Albarracin and Valter, 2012; Peoples et al., 2012a; Begum et al., 2013; Gkotsi et al., 2014), traumatic brain (Ando et al., 2011; Oron et al., 2012; Quirk et al., 2012; Xuan et al., 2013, 2014, 2015) and optic nerve (Fitzgerald et al., 2010) injury, experimentally induced stroke (Lapchak et al., 2004; DeTaboada et al., 2006; Oron et al., 2006), familial amyotrophic lateral sclerosis (Moges et al., 2009), multiple sclerosis (Muili et al., 2012, 2013), aging (Begum et al., 2013; Kokkinopoulos et al., 2013; Gkotsi et al., 2014; El Massri et al., 2018b), Parkinson’s disease (Liang et al., 2008; Whelan et al., 2008; Ying et al., 2008; Trimmer et al., 2009; Shaw et al., 2010, 2012; Peoples et al., 2012b; Moro et al., 2013, 2014, 2016; Purushothuman et al., 2013; Vos et al., 2013; Johnstone et al., 2014; Reinhart et al., 2014, 2016a,2016b,^2017^; Oueslati et al., 2015; Darlot et al., 2016; El Massri et al., 2016a,b, 2017; 2018a; Kim et al., 2019; O’Brien and Austin, 2019; San Miguel et al., 2019; Salehpour and Hamblin, 2020) and Alzheimer’s disease (Michalikova et al., 2008; Yang et al., 2010, 2021; DeTaboada et al., 2011; Sommer et al., 2012; Grillo et al., 2013; Purushothuman et al., 2014, 2015; Comerota et al., 2017, 2019; Blivet et al., 2018; Wang et al., 2020).

### Mechanisms of photobiomodulation: Direct and indirect stimulations

The precise mechanisms used by photobiomodulation to achieve these beneficial outcomes – both functional and neuroprotective – are not entirely clear, but two main ones have been suggested, namely direct and indirect systemic stimulation (Johnstone et al., 2016; Mitrofanis, 2019).

**FIGURE 1:**
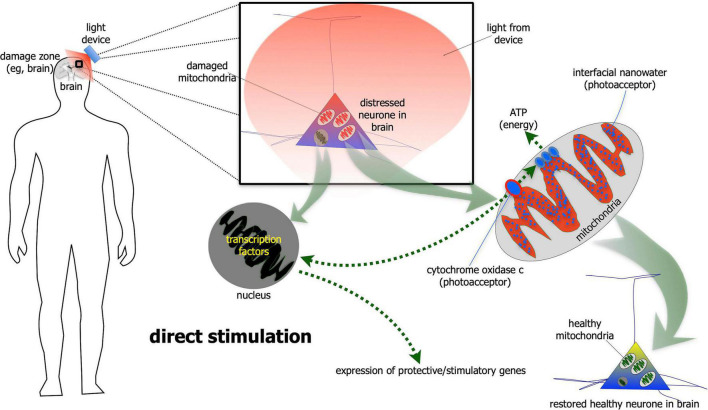
Schematic diagram showing the direct stimulation mechanism of photobiomodulation. When photobiomodulation hits the cells directly, it is absorbed by a photoacceptor, most notably cytochrome oxidase c or interfacial nanowater within the mitochondria. This results in the production of more ATP (adenosine triphosphate) energy and to the activation of transcription factors in the nucleus and expression of protective and stimulatory genes within the cells. The net result is a healthier cell.

For direct stimulation, photobiomodulation has to fall directly on the neurones (Figure 1). The light is absorbed by photoacceptors found among mitochondria (e.g., cytochrome oxidase c and/or interfacial nanowater) or elsewhere (e.g., transient potential receptor ion channels and/or various types of opsins) within the neurones, that then generates more energy that drive intrinsic neuronal functions (Hamblin, 2016; Bathini et al., 2022; Hamblin and Liebert, 2022; Liebert et al., 2022; Ramezani et al., 2022). In addition to these short-term energy gains, photobiomodulation also induces more long-term cellular changes, by activating the expression of various functional and protective genes (Hamblin, 2016). In particular, photobiomodulation prompts the expression of growth factors, for example glial-derived neurotrophic factor (El Massri et al., 2017) and brain-derived neurotrophic factor (Meng et al., 2013; Xuan et al., 2013), both of which have been shown to increase the survival of neurones. In essence, photobiomodulation makes the neurones “healthier,” by not only making them function better, but also making them more resistant to disease and distress (Hamblin, 2016; Mitrofanis, 2019). It should be noted that this direct type of stimulation has been shown to influence both the functional activity of neurones, as well as offer neuroprotection (Hamblin, 2016; Mitrofanis, 2019).

Photobiomodulation not only has a direct neuroprotective effect on diseased or distressed neurones, but it also has an impact on the hypertrophy and proliferation of the resident glial cells and inflammation. Previous studies have shown that photobiomodulation reduces gliosis and/or inflammation in animal models of Alzheimer’s (DeTaboada et al., 2011; Blivet et al., 2018) and Parkinson’s disease (El Massri et al., 2016a,b; O’Brien and Austin, 2019), multiple sclerosis (Muili et al., 2012, 2013), aging (Begum et al., 2013; Kokkinopoulos et al., 2013; Gkotsi et al., 2014; El Massri et al., 2018b) and traumatic brain injury (Khuman et al., 2012). It is not clear if the photobiomodulation-induced reduction in gliosis and/or inflammation is due to a direct action on the glial cells or secondary to the survival of the neurones, although there are reports of a direct photobiomodulation stimulation of primary astrocytes in culture (Yang et al., 2010; Yoon et al., 2021). It should be noted that photobiomodulation has also been shown to have a direct effect on the vascular system. There is evidence that photobiomodulation offsets the degeneration and leakage of retinal capillaries in animal models of diabetes (Cheng et al., 2018) and in the striatum and brainstem of a mouse model of Parkinson’s disease (San Miguel et al., 2019). Further, photobiomodulation has been reported to induce the release of nitric oxide from cells, which triggers the vasodilation of nearby blood vessels, increasing blood (and lymphatic) flow (Hamblin, 2016).

**FIGURE 2:**
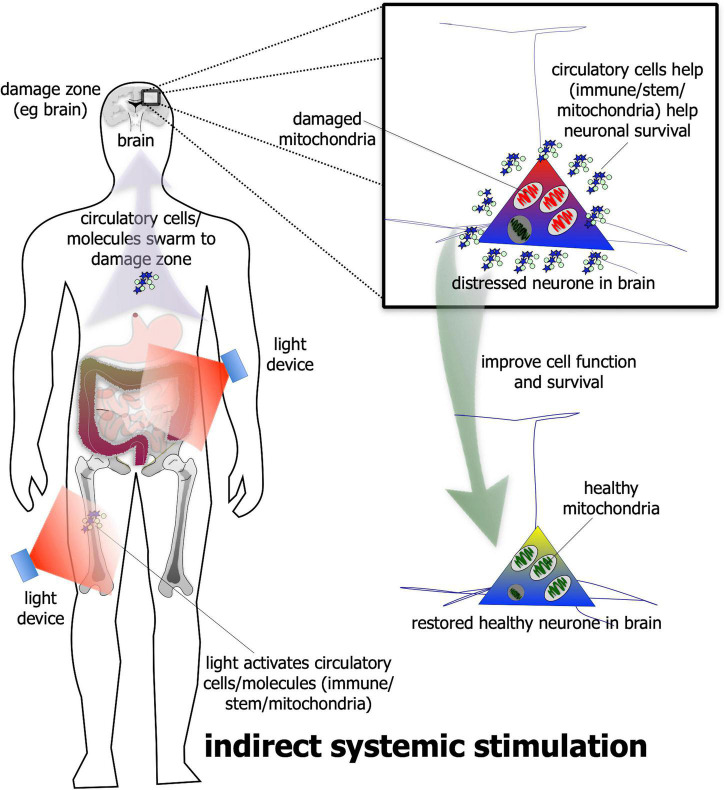
Schematic diagram showing the indirect stimulation mechanism of photobiomodulation. When photobiomodulation is applied to an organ/structure (e.g., thigh or abdomen) distant from the damage zone (e.g., brain), it may activate circulatory cells, for example stem or immune cells or even the free-floating mitochondria in the plasma, that swarm to the site of damage and improve cell function and/or survival. The net result is a healthier cell. Although this method can be effective in neuroprotection in a range of animal models of disease, it is not quite as effective as the direct photobiomodulation stimulation.

In addition to direct stimulation, photobiomodulation has been shown – quite remarkably – to be beneficial to neuronal survival even when it is applied to a distant or remote location; that is, when it is not applied directly to the neurones (Figure 2). The evidence for this indirect stimulation has been accumulated from many previous studies in a range of animal models of disease – from diabetes to Alzheimer’s and Parkinson’s disease – showing that photobiomodulation applied to one body part can induce neuroprotective effects in another, more distant body part (Braverman et al., 1989; Tuby et al., 2011; Stone et al., 2013; Johnstone et al., 2014, 2016; Liebert et al., 2014; Farfara et al., 2015; Saliba et al., 2015; Oron and Oron, 2016; Mitrofanis, 2017; Blivet et al., 2018; Kim et al., 2019). For this effect, photobiomodulation is thought to activate circulating immune (Byrnes et al., 2005; Chung et al., 2012; Muili et al., 2012, 2013; Saliba et al., 2015) and/or stem (Tuby et al., 2011; Arany et al., 2014; Farfara et al., 2015; Khan and Arany, 2015; Oron and Oron, 2016) cells, or even free-floating mitochondria (Al Amir Dache et al., 2020), within the cardiovascular or lymphatic systems that then leads to an increase in overall mitochondrial activity – in a similar fashion to the direct stimulation described above – in the distressed neurones located in the brain. The precise mechanism used by the circulatory cells and/or molecules to achieve these beneficial outcomes in neurones are far from clear, however (Johnstone et al., 2016; Mitrofanis, 2019). Although this type of indirect photobiomodulation stimulation has been shown to be neuroprotective, it is not known if it can induce a functional change in the activity of neurones within the brain, as does the direct stimulation (see above).

In summary, both the direct and indirect types of photobiomodulation stimulation have been reported to be neuroprotective – when applied in the state of wakefulness – in a range of animal models of disease. When comparing the two, at least in an animal model of Parkinson’s disease (Johnstone et al., 2014) – the direct stimulation is more effective than the indirect, resulting in a larger magnitude of neuroprotection. It has been suggested that the direct stimulation forms the primary mechanism of photobiomodulation-induced neuroprotection, while the indirect stimulation forms an added, back-up one (Johnstone et al., 2016; Mitrofanis, 2019).

## Photobiomodulation and sleep

Photobiomodulation in the state of wakefulness has been much studied, whereas the impact of photobiomodulation on brain function during the state of sleep has received little attention. In the section that follows, we will discuss what is known of the effect of photobiomodulation on the sleeping brain, followed by the potential mechanisms involved. First, we will consider the primary function associated with sleep and the recently discovered system that is involved in carrying out this function.

### Glymphatic system: The house-keeper of the sleeping brain

Although the precise function of sleep is not clear, it has been suggested recently to be the critical period of the 24 h cycle when the brain replenishes its resident fluid and clears all of its cellular debris and waste-products before they become toxic; that sleep is when the brain assumes a house-keeping function, a function that it cannot undertake readily during wakefulness, when it is fully occupied with orchestrating all the complex neural networks associated with the executive functions, namely cognition, attention, memory and skilled movements (Iliff et al., 2012; Rasch and Born, 2013; Aspelund et al., 2015; Eugene and Masiak, 2015; Jessen et al., 2015; Louveau et al., 2015, 2017; Brodziak et al., 2018; Plog and Nedergaard, 2018; Hablitz et al., 2020; Mestre et al., 2020; Nedergaard and Goldman, 2020; Reddy and van der Werf, 2020; Yan et al., 2021). Recent studies have reported that both children and adults are able to retain memories better after they slept well. Two naps per day during infancy results in better memory of tasks (Mason et al., 2021) and being kept awake results in a tendency to forget reward memory representations (Prehn-Kristensen et al., 2018).

**FIGURE 3:**
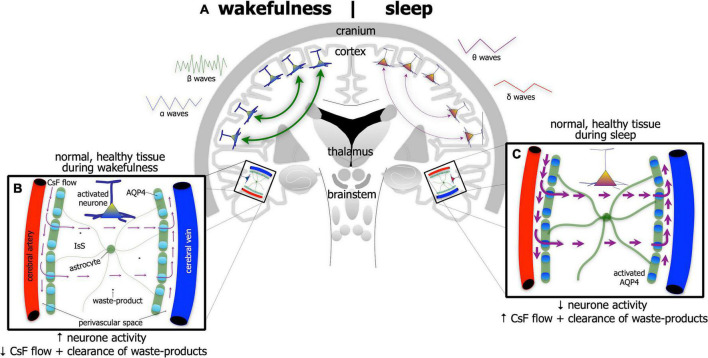
Schematic diagram of the brain **(A)** highlighting the glymphatic system – the house-keeper of the brain – in wakefulness [left side of brain schematic in panel **(A)**; and inset **(B)**] and in sleep [right side of brain schematic in panel **(A)**; and inset **(C)**]. Glymphatic activity is greater during sleep [represented by thicker astrocyte processes and thicker arrows of cerebrospinal fluid (CsF) flow in panel **(C)**] than in wakefulness **(B)**. Its activity is correlated with changes in brain wave-forms in the different arousal states; it increases with the onset and predominance of δ and θ waves, characteristic of slow-wave [right side of panel **(A)**] and reduces with onset and predominance of α and β waves, characteristic of wakefulness [left side of panel **(A)**]. The glymphatic system works with CsF flowing into perivascular spaces around the arteries and then into the interstitial space (IsS) within the brain via aquaporin-4 (AQP4) in astrocytic end-feet. AQP4 is more active during sleep **(C)** than in wakefulness **(B)**. This process drives the drainage of excess fluid and waste into perivascular spaces around the veins.

The brain undertakes its house-keeping duties in a rather unique way. Unlike all other organs, the brain does not have distinct lymphatic vessels and nodes to clear fluid and waste-products into the venous system. Rather, the brain relies on a series of perivascular spaces and astrocytic glial cells to clear its fluid and waste (Figures 3A–C). Cerebrospinal fluid flows into perivascular spaces around the arteries and then into the interstitial space within the brain via water channels (aquaporin-4) in astrocytic end-feet. This process then drives the drainage of excess fluid and waste within the interstitial space out and into perivascular spaces around the veins, disposing ultimately through lymphatic vessels through the meninges and then down into the venous system (Iliff et al., 2012; Rasch and Born, 2013; Aspelund et al., 2015; Eugene and Masiak, 2015; Jessen et al., 2015; Louveau et al., 2015, 2017; Brodziak et al., 2018; Plog and Nedergaard, 2018; Hablitz et al., 2020; Mestre et al., 2020; Nedergaard and Goldman, 2020; Reddy and van der Werf, 2020; Yan et al., 2021).

The activity of the glymphatic system is much greater in the state of sleep (Figure 3C) than in wakefulness (Figure 3B). Its activity has been shown to be correlated tightly with changes in brain wave-forms in the different arousal states; it increases with the onset and predominance of δ and θ waves, characteristic of slow-wave, non-rapid eye movement sleep and reduces with the onset and predominance of α and β waves, characteristic of wakefulness (and rapid-eye movement sleep) (Iliff et al., 2012; Rasch and Born, 2013; Aspelund et al., 2015; Eugene and Masiak, 2015; Jessen et al., 2015; Louveau et al., 2015, 2017; Brodziak et al., 2018; Plog and Nedergaard, 2018; Hablitz et al., 2020; Mestre et al., 2020; Nedergaard and Goldman, 2020; Reddy and van der Werf, 2020; Yan et al., 2021).

**FIGURE 4:**
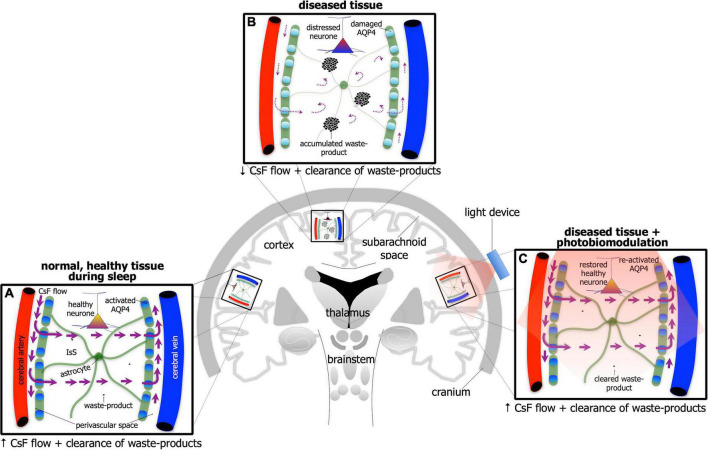
Schematic diagram of the brain highlighting the glymphatic system - the house-keeper of the brain – in normal, healthy tissue during sleep **(A)**, in disease [**(B)**; e.g., Alzheimer’s disease] and in disease after photobiomodulation treatment **(C)**. Glymphatic activity is reduced greatly in disease [represented by thinner astrocyte processes, thinner/curved arrows of cerebrospinal (CsF) flow through interstitial space (IsS), accumulated waste-product and lighter-colored (damaged) aquaporin protein in **(B)** compared to normal **(A)]**. After photobiomodulation treatment, glymphatic activity is restored [represented by thicker astrocyte processes, thicker arrows of CsF flow through IsS, cleared waste-product and darker-colored (re-activated) aquaporin protein in **(C)** as well as neuronal health].

If individuals are deprived of quality sleep, and the brain does not clear its waste effectively, then many negative consequences may develop; for example, individuals become less attentive, have slower cognitive function and memory recall, and/or have problems with motor functions. Executive function and emotional regulation are diminished. And it does not improve the older we get; those over 60 years tend to have shorter and lighter sleep patterns, interrupted often by multiple awakenings. Consistent with these observations, there are many reports of an age-related decline in glymphatic activity, both in cerebrospinal fluid flow and clearance. When periods of poor quality sleep become chronic, there is an increased risk of developing a serious neurological condition, including depression or Alzheimer’s disease. In Alzheimer’s disease for example, there are many reports that the activity of the glymphatic system is very much reduced (Figures 4A,B). In mouse models of the disease, there is reduced glymphatic influx, resulting in less clearance of the β-amyloid (Iliff et al., 2012; Rasch and Born, 2013; Aspelund et al., 2015; Eugene and Masiak, 2015; Jessen et al., 2015; Louveau et al., 2015, 2017; Brodziak et al., 2018; Plog and Nedergaard, 2018; Hablitz et al., 2020; Mestre et al., 2020; Nedergaard and Goldman, 2020; Reddy and van der Werf, 2020; Yan et al., 2021) and tau proteins (Harrison et al., 2020).

### Photobiomodulation influence on the sleeping brain and the house-keeper

No previous study has examined the effect of photobiomodulation on brain wave-forms during sleep; for example, whether it influences the activity of the δ and θ waves during slow-wave sleep, as it does these waves and the α, β and γ waves during wakefulness (Vargas et al., 2017; Wang et al., 2017; Jahan et al., 2019; Zomorrodi et al., 2019). There have, however, been recent indications that photobiomodulation does in fact impact the clearance of fluid and toxic waste more effectively during sleep than during wakefulness (e.g., Semyachkina-Glushkovskaya et al., 2021b).

Transcranial photobiomodulation has been shown to improve the clearance of experimentally introduced substances (e.g., gold nanorods and dextran) into the cerebrospinal fluid (Semyachkina-Glushkovskaya et al., 2020). In addition, photobiomodulation reduces β-amyloid accumulation and the cognitive loss of Alzheimer’s-induced mice more effectively during sleep, than during wakefulness (Semyachkina-Glushkovskaya et al., 2021b). It also stimulates the overall flow of cerebrospinal fluid through the brain, as well as prompting the break-up of β-amyloid aggregations (Figure 4C; Yue et al., 2019). When applied to normal mice at night, photobiomodulation promotes a faster clearance of β-amyloid from the ventricular system of the brain, than when it is applied during the day (Semyachkina-Glushkovskaya et al., 2021b). Finally, photobiomodulation has been shown to stimulate the clearance of fluid from the meningeal lymphatic vessels; these vessels are considered crucial in the final clearance of β-amyloid from the brain (Zinchenko et al., 2019).

### Photobiomodulation-induced mechanisms during sleep

The precise mechanisms used by photobiomodulation to stimulate the activity of the glymphatic system, that is to improve the fluid clearance and disposal of waste-products from the brain, are far from clear (Salehpour et al., 2022). Previous authors have shown that photobiomodulation prompts the breakdown of various protein aggregations within the brain and stimulates a nitric oxide-induced vasodilation – at least outside the brain, within the lymphatic vessels of the meninges – and these are likely to contribute to the improved fluid and waste clearance from the brain (Yue et al., 2019; Semyachkina-Glushkovskaya et al., 2020, 2021a,2021b,2021c). Many other mechanisms, as yet unknown, are, however, likely to be at play also, particular within the glymphatic system.

## A speculation: There is an arousal-dependent effect of photobiomodulation

Taking all these findings together, we suggest that photobiomodulation has different cellular effects, depending on the state of arousal when it is applied, that it may follow a circadian rhythm. During wakefulness, photobiomodulation could have a dual primary effect of first, stimulating neuronal function, activating mitochondrial activity and gene expression, and influencing the different wave-forms patterns across the brain (e.g., α, β, γ waves), and second, improving neuronal survival, providing effective neuroprotection against distress and disease. During sleep, however, photobiomodulation may be less effective in stimulating neuronal function and survival, but be more effective in stimulating the clearance of fluid and waste from the brain; it may do so by increasing the activity of the glymphatic system. The mechanism that underpins this glymphatic stimulation is not known, but we suggest that photobiomodulation may work primarily to increase the permeability of the aquaporin-4 water channels on the astrocytes, thereby helping to increase the flow of fluid through the brain (Figure 4C). This suggestion requires experimental validation by future studies, however. Semyachkina-Glushkovskaya and colleagues suggest that the fluid clearance of the brain is promoted further by photobiomodulation-induced vasodilation of the meningeal lymphatic vessels, and this mechanism may help the process also (Semyachkina-Glushkovskaya et al., 2020, 2021a,2021b,2021c). Further, photobiomodulation may also effect the composition of cerebrospinal fluid, by changing the structure of the water molecules, making the cerebrospinal fluid more free flowing (Salehpour et al., 2022). In addition, the flow of the cerebrospinal fluid may be influence by the cilia lining the ventral parts of the third ventricle which are considered to be under circadian control (Eichele et al., 2020); photobiomodulation may have a considerable influence this system as well.

Our suggestion of an arousal-dependent effect of photobiomodulation is not inconsistent with recent findings that photobiomodulation influences cell function differently depending on the time of day when it is applied, that photobiomodulation-induced cellular effects follow circadian rhythms. In a drosophila model, photobiomodulation has been reported to increase mitochondrial function and ATP (adenosine triphosphate) levels more effectively in the mornings, compared to the afternoons or at night (Weinrich et al., 2019; Shinhmar et al., 2022). There are also indications of a similar pattern in humans, that photobiomodulation improves visual function more effectively when applied in the mornings compared to later in the day, in the afternoons (Shinhmar et al., 2020). Hence, it appears that as the day proceeds from morning to night, the effect of photobiomodulation on mitochondrial function becomes less; as suggested above, the photobiomodulation impact on other cellular structures, such as the water channels on astrocytes may become stronger during the shift from day to night.

The speculation outlined above, that there is an arousal-dependent effect of photobiomodulation on different cell types and systems in the brain relates to a direct stimulation (Figure 1). But what about indirect systemic stimulation (Figure 2)? Does photobiomodulation have a different effect on circulatory cells and/or molecules in the different states of arousal, that it could also follow a circadian rhythm? It may be the case for example, that photobiomodulation has less of an effect on the recently discovered free-floating plasma mitochondria during sleep than during wakefulness. Further, for the immune system, photobiomodulation during wakefulness may promote the prevalence of anti-inflammatory cytokines (Muili et al., 2012, 2013), while during sleep, it may enhance defense mechanisms against infection and inflammation, with the production of pro-inflammatory cytokines (Besedovsky et al., 2012; Garbarino et al., 2021; Mapunda et al., 2022). These key issues for indirect photobiomodulation stimulation during different arousal states remain to be determined.

## A speculation: “Biophotons” contribute to the mechanism of photobiomodulation and the arousal-dependent effect

A question that is asked commonly by scientists and by those across the wider community is “why do neurones located so deep within brain – those that are not normally exposed to light and function in total darkness – have light-sensitive receptors?” It stands to reason that cells of the skin for example, have light-sensitive receptors as to facilitate the production of vitamin D, but why should cells located very deep in the brain have receptors to light?

One could always use the evolutionary argument that, all cells – even those found deep within the brain – have maintained light-sensitive receptors as a remnant from a simpler invertebrate ancestor, when all cells were exposed directly to light and they were in a position to convert light energy into metabolic energy. If so, this would explain why modern day photobiomodulation is so effective (e.g., Mitrofanis, 2017). But is there more to the story? We suggest that there is. That neurones, in fact all body cells, have light-receptors because they themselves use light to communicate with each other; they also use light to repair themselves, as well as others, during periods of distress and/or damage. In essence, we suggest that photobiomodulation is effective on neuronal function and survival because neurones use the very same wavelengths to communicate and for repair (Liebert et al., 2014; Moro et al., 2021). In the section that follows, we will consider what is known of how neurones may generate light and then highlight the idea that photobiomodulation uses this light system to impart beneficial outcomes; we speculate further that the change in photobiomodulation effects depending on the state of arousal is reflective of a change in biophoton activity from wakefulness to sleep.

### Biophotons: The light made by cells

The idea that all living cells can generate light and may use this to communicate with each other is not new, having been first proposed about a century ago. Since that time, the evidence for this form of communication has developed further and has been referred to as biophotons. This self-generated light is thought to arise from the many intrinsic metabolic processes that occur within the cell, principally from the mitochondria, and be absorbed by a number of chromophores (e.g., cytochrome oxidase c) either within the same or neighboring (i.e., bystander) cells, leading ultimately to a change in electrical activity (Grass et al., 2004; Tang and Dai, 2014; Salari et al., 2015; Mothersill et al., 2019; Van Wijk et al., 2020; Moro et al., 2021; Zangari et al., 2021).

**FIGURE 5:**
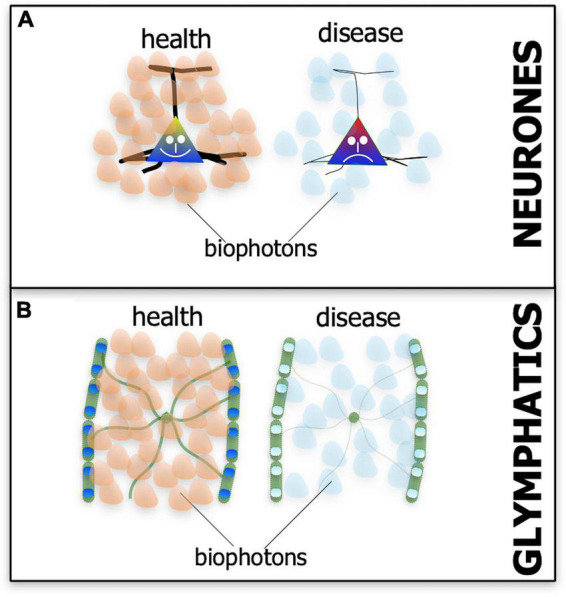
Schematic diagram showing the suspected patterns of biophoton emissions – the endogenous light from cells – in neurones **(A)** and the glymphatic system **(B)** in both health (A’,B’) and disease (A”,B”). In health, the biophoton light emitted by neurones and glymphatics may be different in terms of wavelength and intensity to that emitted in disease (represented by different-colored and fewer biophoton shapes).

Biophotons are not bright, hence their often used alternative term, ultra-weak light emissions. They cannot be seen by the naked eye, nor even a fluorescence microscope, but only with an ultra-sensitive light detection device or with a very specific histological stain. The biophoton emissions have a rather broad range of wavelengths, from ultraviolet to red and near infrared range (i.e., λ = 200–950 nm), the latter being within the range of photobiomodulation (Dotta et al., 2014; Tang and Dai, 2014; Zangari et al., 2021). It is not clear whether biophoton emissions from the mitochondria initially formed by accident, as a byproduct of metabolic activity, or by design, serving a specific purpose. Either way, all neurones may have evolved the biophoton network to communicate and for repair (Grass et al., 2004; Tang and Dai, 2014; Salari et al., 2015; Mothersill et al., 2019; Van Wijk et al., 2020; Moro et al., 2021; Zangari et al., 2021). A most striking feature of biophotons emissions from cells is that they can vary – in terms of intensity and wavelength - depending on the state of homeostasis, whether the cell is healthy or diseased (Figure 5; Tang and Dai, 2014; Salari et al., 2015).

### Does photobiomodulation engage the biophoton network of communication and repair?

We have speculated previously that many of the beneficial effects on cell function and survival provided by photobiomodulation may in fact depend on the biophoton network (Liebert et al., 2014; Moro et al., 2021). It is striking that the range of the wavelengths used by biophotons (∼λ = 200–950nm) overlaps with the range of wavelengths effective in photobiomodulation (∼λ = 600–1000), albeit at much lower intensities, and that the same organelles (i.e., mitochondria) and chromophores (e.g., cytochrome oxidase c) have been implicated in both. The idea also offers an explanation as to why neurones located so deep within the near total-darkness of the brain, have receptors to light and benefit from photobiomodulation. That is because they themselves use light to communicate and maintain homeostasis and photobiomodulation engages this network system to produce beneficial outcomes (Liebert et al., 2014; Moro et al., 2021).

This speculation, that photobiomodulation works through the biophoton network to achieve beneficial effects, is based on a direct stimulation (Figure 1). The benefits of photobiomodulation using the indirect systemic stimulation (Figure 2) may rely on the biophoton network also. The free-floating mitochondria within the blood plasma are of particular interest here. They too could use biophotons to communicate and repair, as they would as intracellular organelles, so there is every possibility that they may be activated by photobiomodulation – particularly during the state of wakefulness – and swarm to the site of distress, helping neurones survive with their biophoton emissions. It remains to be determined experimentally by future studies if the biophoton network is in fact engaged by photobiomodulation, either by direct or indirect means, and whether this is indeed the major mechanism that underpins the beneficial outcomes of photobiomodulation.

### Photobiomodulation and the arousal-dependent effect: Reflective of a change in biophoton activity?

**FIGURE 6:**
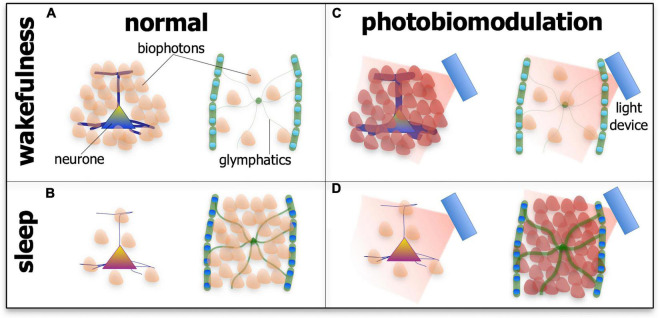
Schematic diagram showing the suspected patterns of biophoton emissions – the endogenous light from cells – in neurones and the glymphatic system during wakefulness **(A,C)** and sleep **(B,D)** and in normal tissue **(A,B)** and after photobiomodulation treatment. During wakefulness in normal tissue **(A)**, the activity of neurones is high (represented by thick processes and many biophoton shapes), while glymphatic activity is low (represented by thin processes and few biophoton shapes). During sleep in normal tissue **(B)**, the activity of neurones is low (represented by thin processes and few biophoton shapes), while glymphatic activity is high (represented by thick processes and many biophoton shapes). After photobiomodulation treatment, activity is increased further in neurones during wakefulness [**(C)**; represented by thick processes and many darker biophoton shapes] and in the glymphatics during sleep [**(D)**; represented by thick processes and many darker biophoton shapes]. Photobiomodulation may have less of an effect on neurones during sleep [**(D)**; represented by thin processes and few biophoton shapes] and on the glymphatics during wakefulness [**(C)**; represented by thin processes and few biophoton shapes].

If this speculation is correct, that photobiomodulation engages the biophoton network to achieve beneficial outcomes in neuronal function and survival, then the arousal-dependent changes in the effect of photobiomodulation may reflect changes in biophoton activity in the different states. We speculate further that the most functionally active cells or systems during either wakefulness or sleep would be the most active and receptive in biophoton transmissions, thereby being the most responsive to photobiomodulation (Figure 6). During wakefulness, glymphatic activity is low, while many neurones across the brain have high metabolic activity and presumably high biophoton activity, both in emission and reception; as a consequence, photobiomodulation would be most effective on these cells during this state. During sleep, however, many neurones are at rest, while the glymphatic system becomes active, in particular the astrocytes and their aquaporin-4 water channels, and presumably has high biophoton activity; hence, photobiomodulation would be most effective on this system during this state of arousal (Figure 6).

## Conclusion

In most studies reporting on the cellular and clinical effects of photobiomodulation, the treatment has been applied during the state of wakefulness. These studies have shown that photobiomodulation improves neuronal function and survival in the brain after stimulating mitochondrial activity in neurones, as well as activating a range of stimulatory and protective pathways; they also show improvements in clinical signs and/or symptoms in a range of disorders, from Alzheimer’s to Parkinson’s disease, and from depression to traumatic brain injury. Many fewer studies have examined the effect of photobiomodulation delivered during sleep. These few studies have nevertheless shown a somewhat different effect on brain function. In this state, photobiomodulation appears to be more effective in improving the flow of cerebrospinal fluid and clearance of waste from the brain. We speculate that the overall effects of photobiomodulation on the brain are arousal-dependent, shifting from different cells and systems as wakefulness becomes sleep, that it may follow a circadian rhythm. We speculate further that the different arousal-dependent effects of photobiomodulation are mediated principally through the biophoton – ultra-weak light emission – network of communication and repair across the brain. If our speculations are correct, then this shift in beneficial effects induced by photobiomodulation – dependent on the state of arousal – have considerable experimental and therapeutic implications. Our speculations on the effects of photobiomodulation on the glymphatic system, in particular its impact on the activity of aquaporin-4 water channels, as well as the biophoton network await experimental validation by future studies.

## Author contributions

All authors contributed to the writing and editing of the manuscript.

## Conflict of interest

CH was a director and co-founder of the WellRed coronet helmet. The remaining authors declare that the research was conducted in the absence of any commercial or financial relationships that could be construed as a potential conflict of interest.

## Publisher’s note

All claims expressed in this article are solely those of the authors and do not necessarily represent those of their affiliated organizations, or those of the publisher, the editors and the reviewers. Any product that may be evaluated in this article, or claim that may be made by its manufacturer, is not guaranteed or endorsed by the publisher.
